# Comprehensive review of recent advances in Pompe disease: pathogenesis, management, and future directions

**DOI:** 10.3389/fneur.2026.1756935

**Published:** 2026-02-17

**Authors:** Guangrui Li

**Affiliations:** 1 Department of Neurology, The First Hospital of Hebei Medical University, Shijiazhuang, China; 2 Neuromedical Technology Innovation Center of Hebei Province, Shijiazhuang, China; 3 Department of Infectious Diseases, The Third Hospital of Hebei Medical University, Shijiazhuang, China

**Keywords:** alglucosidase alfa, avalglucosidase alfa, cipaglucosidase alfa, digital health technology, enzyme replacement therapy, glycogen storage disease type II, newborn screening, Pompe disease

## Abstract

Pompe disease is an autosomal recessive lysosomal storage disorder caused by deficiency of acid alpha-glucosidase (GAA), leading to pathological glycogen accumulation in multiple tissues. This review synthesizes recent progress in understanding and managing Pompe disease, encompassing advances in newborn screening (NBS), novel biomarkers, next-generation enzyme replacement therapies (ERTs), gene therapy, and digital health technologies (DHTs) for monitoring. We also examine the associated economic burden and mortality patterns. Next-generation ERTs, including avalglucosidase alfa and cipaglucosidase alfa combined with miglustat, have improved outcomes and safety. Emerging strategies like transferrin receptor-mediated ERT and muscle-targeted adeno-associated virus (AAV) vectors show promise for overcoming current limitations, including central nervous system (CNS) involvement. DHTs enable sensitive detection of motor impairment even in presymptomatic stages. Despite progress, challenges remain in early detection, long-term management, and healthcare resource allocation. Future success requires integrated strategies combining NBS, innovative therapeutics, sensitive monitoring, and supportive policies.

## Introduction

Pompe disease, or glycogen storage disease type II (GSDII), is caused by mutations in the acid alpha-glucosidase *(GAA)* gene leading to deficient acid alpha-glucosidase activity, impairing lysosomal glycogen breakdown (1, 2). This enzymatic defect leads to pathological glycogen accumulation primarily in skeletal, cardiac, and smooth muscles. The disease spectrum ranges from severe infantile-onset Pompe disease (IOPD), characterized by cardiomyopathy and rapid progression, to more heterogenous late-onset Pompe disease (LOPD) with predominant skeletal and respiratory muscle involvement (3, 4). Beyond lysosomal pathology, mitochondrial dysfunction is increasingly recognized as a central mechanism in disease complications. Glycogen accumulation disrupts autophagic flux, impairing the clearance of damaged mitochondria (mitophagy), leading to oxidative stress and bioenergetic failure. This contributes to disease severity and explains why current ERTs, which do not cross the blood-brain barrier (BBB), fail to address neurological decline (5).

The introduction of firstgeneration enzyme replacement therapy (ERT) with alglucosidase alfa (ALG, Myozyme^®^ in 2006/Lumizyme^®^ in 2010) transformed management, especially for IOPD (6, 7). The second-generation of ERT was avalglucosidase alfa (AVA, Nexviazyme^®^/Nexviadyme^®^), which was approved in 2021. AVA showed enhanced M6P targeting for better muscle uptake superior respiratory function improvements compared to 1st generation. The third-generation ERT was Cipaglucosidase alfa + Miglustat (Cipa+mig, Pombiliti^®^ + Opfolda^®^), which was a combination therapy approved in 2023 with enzyme stabilization technology, offering an alternative for antibody-positive LOPD patients (8, 9). However, challenges like gradual functional decline, treatment resistance, high costs, and untreated CNS manifestations persist.

This review consolidates recent evidence on the natural history, economic impact, novel biomarkers, evolving treatments, animal model, induced stem cell, and the roles of NBS and DHTs. We integrate three critical emerging areas: (1) the application of DHTs for pre-symptomatic motor impairment detection, (2) the potential of transferrin receptor-mediated ERT to address CNS disease, and (3) a critical appraisal of cost-effectiveness modeling challenges when standard care is itself cost-ineffective. These perspectives provide a forward-looking framework for research and policy.

## Newborn screening: evolution and implementation

Early diagnosis is crucial for initiating ERT before irreversible muscle damage occurs. Traditional newborn screening (NBS) utilizing heel-prick dried blood spots (DBS) faces logistical challenges, particularly with early hospital discharge. Cheah et al. (10) validated umbilical cord blood (UCB) as a viable alternative.

In their analysis of 4,091 UCB samples, mean GAA activity was comparable to Day 3 heel-prick samples from a Taiwanese cohort in Malaysia. Applying a cutoff of 1.54 μM/h (0.1% of the population), one confirmed Pompe case was identified(prevalence of 1 in 4,091, or 0.024%) suggesting possible regional variations or underdiagnosis. The identified infant, compound heterozygous for a novel deletion (c.2005_2010del) and a known likely pathogenic variant (c.1123C>T), was clinically asymptomatic at 3 months, consistent with LOPD (10).

UCB GAA activity remained stable for 72 h at 4 °C. Pseudodeficiency alleles posed a challenge, with 12 of 13 initially flagged infants carrying benign variants (e.g., c.1726G>A) that reduce GAA activity without causing disease. This underscores the need for a two-tiered strategy (enzyme activity followed by genetic confirmation). UCB sampling is particularly useful for regions with high hospital turnover or limited community infrastructure. These advances in early diagnosis set the stage for understanding how treatment timing influences long-term outcomes (10).

## Systematic assessment of enzyme replacement therapies

Corbett et al. (11) conducted a systematic review and network meta-analysis (NMA) evaluating ALG, AVA, and Cipa+mig vs. each other and best supportive care (BSC).

The review included 38 studies (3 RCTs, 3 extension studies, 7 registry studies, 25 single-group prospective studies). For ERT-naïve patients at ~1 year, both ALG and AVA showed statistically significant 6MWT improvements vs. BSC (mean differences ≈25m and ≈54m, respectively). Cipa+mig showed numerical superiority not reaching statistical significance, likely due to small ERT-naïve patient numbers in PROPEL. For FVC % predicted, all three ERTs showed numerical superiority over BSC without reaching statistical significance.

Direct and indirect comparisons suggested potential AVA advantage over ALG in 6MWT, statistically significant in primary analysis but attenuated to non-significant in sensitivity analysis adjusting for COMET trial data skewness (11). The review highlighted evidence limitations: high risk of bias in two pivotal RCTs (LOTS and PROPEL) due to selective outcome reporting, inability to access individual participant data, and scarce long-term comparative evidence (Table 1).

**Table 1:** Next-generation enzyme replacement therapies for late-onset Pompe disease.

**Therapy**	**Mechanism of action**	**Key clinical trial data**	**Safety considerations**
AVG	Recombinant human GAA with ≈15-fold higher M6P content for enhanced CI-MPR receptor binding and cellular uptake.	COMET trial: Superiority to ALG in respiratory function; maintained efficacy through 145 weeks (19, 21).	Favorable safety profile; decreased immunogenicity over time; similar infusion-associated reaction profile to ALG.
Cipa+mig	Cipa: rhGAA with bis-M6P-enriched N-glycan profile; Mig: enzyme stabilizer.	PROPEL trial: Non-inferiority to ALG; significant biomarker reductions; maintained efficacy through 104 weeks (8, 9, 14).	Boxed warning for hypersensitivity reactions, infusion-associated reactions, risk of cardiorespiratory failure; contraindicated in pregnancy.

This table compares the mechanism of action, pivotal clinical trial data, and primary safety considerations for avalglucosidase alfa and cipaglucosidase alfa plus miglustat. Key efficacy data from the COMET and PROPEL trials are summarized, alongside important safety profiles, including immunogenicity and boxed warnings. Populations: adults with LOPD. Study durations: COMET (145 weeks), PROPEL (104 weeks). Outcome domains: respiratory function (FVC), walking ability (6MWT), biomarker reduction (Hex4), safety. M6P, mannose-6-phosphate; CI-MPR, cation-independent mannose-6-phosphate receptor; rhGAA, recombinant human acid alpha-glucosidase.

## Guideline perspectives: two-component therapy

While ERT has improved survival, long-term real-world data show many LOPD patients experience gradual motor and respiratory decline after several years (12). This has driven the development of next-generation ERTs. Cipa+mig is a novel two-component therapy designed for enhanced cellular uptake and enzyme stabilization (13).

The American College of Medical Genetics and Genomics (ACMG) recently published a therapeutics bulletin on Cipa+mig for adult LOPD patients. Cipaglucosidase alfa is distinguished by a cell-line selected N-glycan profile rich in bis-mannose-6-phosphate (bis-M6P), enhancing receptor binding affinity and glycogen clearance. Miglustat, an iminosugar, stabilizes cipaglucosidase alfa during circulation (14). Approval was based on the PROPEL trial and its extension. While the primary endpoint (6MWT improvement at 52 weeks) did not achieve statistical superiority, clinically meaningful improvement was observed in sitting FVC, with statistically significant reductions in creatine kinase and Hex4 compared to AVG (14, 15). Extension data indicated maintained walking distance and stabilized pulmonary function through 104 weeks (14, 16). Evidence is emerging from more severely affected populations previously excluded from pivotal trials. Case series show that non-ambulatory patients transitioning from high-dose ALG can achieve sustained improvements in upper limb strength and biomarkers over 54 months with Cipa+mig, highlighting its potential in advanced disease (17).

In ERT-naïve patients (*n* = 123), AVA demonstrated numerically superior efficacy at 49–52 weeks: differences favored AVA by 4.69–5.49% for predicted forced vital capacity (FVC) and 41.88–57.08 meters for the 6-min walk test (6MWT). The 6MWT difference (57.08 m) reached statistical significance (*P* < 0.02) and is considered clinically meaningful (8, 9, 18). In ERT-experienced patients, AVA showed numerical superiority across outcomes, with maximal expiratory pressure (MEP) improvement (8.62%) reaching significance (18).

Long-term efficacy and safety of AVA are supported by COMET trial 145-week extension data. In this Phase 3 trial, 100 ERT-naïve LOPD patients were randomized to AVA or ALG for 49 weeks, followed by open-label AVA through week 145. 88 patients completed ≥145 weeks. Patients on continuous AVA maintained or improved least squares mean FVC % predicted (+1.38) and 6MWT distance (+20.65 m). Patients switching from ALG to AVA showed further improvement in respiratory and some physical function measures from week 49 to 145. Safety analysis revealed no new concerns, with decreasing anti-drug antibody titers over time. These data support AVA as a first-line treatment and demonstrate the safety and efficacy of switching from ALG to AVA (Table 2) (19).

**Table 2:** Evidence summary for approved enzyme replacement therapies in late-onset Pompe disease.

**Parameter**	**AVG**	**AVA**	**Cipa+mig**
Mechanism	Recombinant human GAA	Recombinant human GAA with enhanced M6P content	bis-M6P-enriched rhGAA + enzyme stabilizer
Pivotal trial	LOTS (6)	COMET (8)	PROPEL (8)
Primary endpoint (6MWT)	+25.1 m vs placebo (*P* = 0.03)	+30.0 m vs ALG (*P* = 0.06)	+21.0 m vs ALG (*P* = 0.07)
Respiratory function (FVC)	FVC: +1.2% vs placebo (*P* = 0.52)	FVC: +2.9% vs ALG (*P* = 0.03)	FVC: +2.5% vs ALG (*P* = 0.06)
Long-term data	Registry data to 13 years (12)	145–week extension (19)	104-week extension (9)
Safety profile	Established long-term safety.	Comparable to ALG.	Boxed warnings; pregnancy contraindication.

A comparative overview of alglucosidase alfa, avalglucosidase alfa, and cipaglucosidase alfa plus miglustat, detailing their mechanisms, pivotal trials, primary and respiratory efficacy endpoints, available long-term data, and safety profiles. Populations: adults with LOPD. Study durations: LOTS (78 weeks), COMET (145 weeks), PROPEL (104 weeks). Outcome domains: walking ability (6MWT), respiratory function (FVC), long-term safety. 6MWT, 6-min walk test; FVC, forced vital capacity.

Safety requires particular attention: a Boxed Warning for hypersensitivity reactions (incidence 27%, 3% severe), infusion-associated reactions (32%), and risk of acute cardiorespiratory failure (14). It is contraindicated in pregnancy due to embryo-fetal toxicity. The ACMG bulletin positions this therapy for adult LOPD patients not improving on current ERT (14). This guidance is complemented by recent perspectives on next-generation therapies, including Singh et al.'s (20) analysis of cipaglucosidase alfa-atga, which unveiled new horizons in Pompe disease therapy.

## Switching from alglucosidase alfa to avalglucosidase alfa: impact on respiratory function

A *post hoc* analysis of the COMET trial provides further evidence supporting the switch from ALG to AVA, regardless of prior response to ALG (21). This analysis, while valuable, carries inherent limitations of retrospective subgroup analyses. It focused on 44 participants who switched from ALG to AVA after week 49. Participants were stratified by their change in upright FVC % predicted (ΔFVC) at Week 49 (thresholds >0% and ≥3%).

Among those who improved on ALG (ΔFVC > 0%, *n* = 20), FVC was maintained after switching to AVA (slope 0.1 %/year, *P* = 0.86). Among those who declined on ALG (ΔFVC ≤ 0%, *n* = 24), FVC stabilized after switching (slope 0.3 %/year, *P* = 0.70), halting the progressive decline observed earlier (slope−3.4 %/year, *P* < 0.01). Similar patterns were observed for FEV1, maximal inspiratory pressure (MIP), and MEP. A sustained reduction in urinary hexose tetrasaccharide (Hex4), a glycogen-derived biomarker, followed the switch, supporting AVA's enhanced cellular uptake (14). These findings indicate switching to AVA can provide meaningful respiratory stabilization for at least 2 years, irrespective of prior ALG response (21).

## Novel pharmacological strategies: enzyme stabilizers

Beyond next-generation ERTs, small-molecule pharmacological chaperones and stabilizers represent a complementary therapeutic strategy. These compounds bind directly to GAA, enhancing stability, cellular uptake, and lysosomal delivery.

Li et al. (22) designed and synthesized novel polyhydroxylated azabicyclo [3.3.1] nonane derivatives (bridged bicyclic iminosugars). The lead candidate, iminosugar 15, demonstrated potent a competitive GAA inhibition (Ki = 0.7 μM) with high selectivity over other glycosidases. As an enzyme stabilizer, it increased GAA melting temperature by 13.3 °C and prevented heat-induced enzyme inactivation, preserving approximately 80% of GAA activity. In Pompe fibroblasts, co-treatment with iminosugar 15 and recombinant GAA resulted in a 3.7-fold increase in intracellular GAA activity, significantly outperforming other derivatives and demonstrating efficacy comparable to N-butyl-deoxynojirimycin (NB-DNJ) (22). This enhancement was mediated through the Mannose-6-Phosphate Receptor pathway, confirming legitimate lysosomal delivery. Bridged bicyclic iminosugars thus represent a promising class of stabilizers for adjunct use with ERT or as standalone chaperones for specific *GAA* mutations.

## Gene therapy advances

Gene therapy represents a promising one-time curative approach. Systemic delivery of adeno-associated virus (AAV) delivery has been limited by the high doses required for effective skeletal muscle targeting, causing liver toxicity and immune responses.

Sellier et al. (23) combined engineered myotropic AAV capsids with a muscle-specific expression cassette. This approach demonstrated superior skeletal muscle targeting and robust liver detargeting (>300-fold reduction vs. AAV9) in *Gaa*^−^/^−^ mice. Enhanced *GAA* expression and glycogen clearance were achieved at doses 10-fold lower than typical AAV9 clinical doses. However, this highly specific muscle targeting elicited a robust and sustained anti-hGAA antibody response in immunocompetent mice, highlighting that *GAA* hepatic expression promotes immune tolerance (23). Future optimized muscle-directed gene therapies may require balancing high muscle specificity with immunogenicity management strategies, potentially via transient immunomodulation or minimal residual liver expression.

## Next-generation delivery systems: transferrin receptor-mediated ERT

A fundamental limitation of current ERTs is their inability to cross the blood-brain barrier (BBB), leaving central nervous system (CNS) manifestations untreated.

George et al. (5) developed a strategy leveraging transferrin receptor (TfR) for enzyme delivery. Fusion proteins combining anti-TfR antibody with recombinant human GAA (anti-TfR-GAA) were evaluated in humanized TfR Pompe mice. A single dose of the lead candidate (FabFc-GAA2) resulted in a 68% reduction in brain glycogen and significant spinal cord clearance—unattainable by standard ERTs. 4 weekly doses normalized CNS glycogen, reduced neuroinflammation, and cleared muscle glycogen more effectively than AVA.

The mechanism involves TfR-mediated transcytosis across the BBB and enhanced muscle uptake, bypassing the CI-MPR pathway. The study also introduced cerebrospinal fluid Hex4 as a potential CNS biomarker and Glyco-CEST/NOE MRI for non-invasive brain glycogen quantification (22). This TfR-mediated approach represents a paradigm shift, potentially addressing both neurogenic and myogenic disease aspects (5).

## Preclinical models: insights from animal and iPSC studies

Extensive preclinical research utilizing *GAA*-knockout mice and patient-derived induced pluripotent stem cells (iPSCs) has been instrumental in elucidating disease mechanisms and testing therapies. A comprehensive summary of key models, their genetic backgrounds, and main findings regarding systemic and tissue-specific pathology (including cardiac, skeletal muscle, nervous system, and smooth muscle involvement), therapeutic responses (to ERT, gene therapy, small molecules), and emerging aspects like mitochondrial dysfunction and autophagy defects is provided in Tables 3, 4. These models consistently replicate core disease features—lysosomal glycogen accumulation, autophagic buildup, mitochondrial abnormalities, and tissue dysfunction—providing validated platforms for mechanistic studies and therapeutic screening. Notably, they highlight challenges like the resistance of type II muscle fibers to ERT, the critical role of immune tolerance in gene therapy, and the involvement of CNS and smooth muscle pathology.

**Table 3:** Extended summary of preclinical animal models in Pompe disease research.

**Animal model**	**Target organ/tissue**	**Mutation**	**Main finding**	**Reference**
*GAA*–KO mouse	Systemic.	Insertional knockout in exon 13	Cardiac hypertrophy. Glycogen-containing lysosomes are detected in liver, heart and skeletal muscle cells.	(32)
*GAA*–KO mouse	Systemic.	Insertional knockout in exon 13	Acid alpha-glucosidase deficiency on a 129xC57BL/6 background resulted in a severe phenotype with progressive cardiomyopathy and profound muscle wasting similar to that in patients with glycogen storage disease type II. On a 129/C57BL/6xFVB background, homozygous mutants developed a milder phenotype with a later age of onset.	(33)
*GAA*–KO mouse	Systemic	*Atg5* gene KO in skeletal muscle on *GAA* KO back-ground.	Lower back muscle weakness, paralysis of hind limbs, muscle atrophy and distinct kyphosis. Accumulation of lysosomal glycogen and decreased autophagy in skeletal muscle. Buildup of endocytic vesicles in the core of fast muscle fibers with accumulation of ubiquitinated (Ub) proteins and P62/SQSTM1.	(34)
*GAA*–KO Mouse	Systemic.	insertional knockout in exon 13	Muscular atrophy and autophagic buildup in muscle fiber with lysosomal enlargement and accumulation of ubiquitinated proteins in the autophagic areas. Actin and myosin filaments, normally arranged in hexagonal arrays, were disordered.	(35)
Alb–T–*hGAA*/–/– mouse; Mck–T–*hGAA*/–/– mouse	Very low *hGAA* expression in all tissue; Restricted expression in skeletal muscle.	Alb–T–*hGAA*/–/–; Mck–T–*hGAA*/–/–;	Low levels of transgene–encoded human *GAA* expressed in skeletal muscle or liver dramatically blunt or abolish the immune response to ERT of hGAA.	(36)
*GAA*–KO mouse	Systemic.	Insertional knockout in exon 13	Gene transfer using an adeno–associated virus vector 2/9 encoding *hGAA* driven by the desmin promotor (rAAV2/9–DES–*hGAA*) induces High GAA Activity and Glycogen Clearance in *GAA*^−/−^ Gastrocnemius.	(37)
Alb–T–*hGAA*/–/– mouse;	Very low hGAA expression in all tissue;	Alb–T–*hGAA*/–/–;	Myoblasts from GAA knock–out mice showed a dramatic expansion of vesicles of the endocytic/autophagic pathways, decreased vesicular movement in overcrowded cells, and an acidification defect in a subset of late endosomes/lysosomes. Type II fibers contain large regions of autophagic buildup that span the entire length of the fibers.	(38)
Alb–T–*hGAA*/–/– mouse;	Very low hGAA expression in all tissue;	Alb–T–hGAA/–/–;	When *GAA* KO mice were treated with rhGAA-/-, skeletal muscle cells took up little enzyme compared to liver and heart. Glycogen clearance was more pronounced in type 1 fibers, and histochemical analysis suggested an increased mannose−6–phosphate receptor immunoreactivity in these fibers.	(39)
Alb–T–*hGAA*/–/– mouse;	Very low hGAA expression in all tissue;	Alb–T–*hGAA*/–/–;	rhGAA efficiently clear glycogen from cardiac muscle and type I muscle fibers, but not type II fibers in skeletal muscle. Low abundance of proteins involved in endocytosis and trafficking of lysosomal enzymes combined with increased autophagy in type II fibers may explain the resistance to therapy.	(40)
*GAA*–KO mouse	Systemic.	Insertional knockout in exon 13	Age–dependent Autophagic Build–up in ERT Resistant Type II Fibers in KO mice. Endocytosed rhGAA is trapped and not processed in the autophagic/lysosomal areas in KO fibers.	(41)
*Gaa* KO^DBA2/J^ mouse	Systemic.	GAA and *Ltbp4* double KO.	Male *GAA* KO^DBA2/J^ mice present most of the key features of the human disease, including early lethality, severe respiratory impairment, cardiac hypertrophy and muscle weakness. Respiratory and muscle function in the *GAA* KO^DBA2/J^ model were significantly improved upon gene therapy with AAV vectors expressing secGAA.	(42)
*GAA*–KO mouse	Systemic.	Insertional knockout in exon 13	Glycogen accumulation also occurs in the mouse nervous system. Gene therapy intracerebroventricular injection of a viral vector carrying *GAA* cDNA increased GAA activity and myelination, and decreased glycogen content and astrogliosis in the brain and spinal cord.	(43)
*GAA*–KO mouse	Systemic.	Insertional knockout in exon 13	*Atg7*, an autophagy gene, was inactivated in muscle of *GAA*-KO Mouse. Suppression of autophagy alone reduced the glycogen level by 50–60%. Following ERT, muscle glycogen was reduced to normal levels, an outcome not observed in Pompe mice with genetically intact autophagy.	(44)
*GAA*–KO mouse	Systemic.	Insertional knockout in exon 13	In *GAA* KO mouse, reduced inter–limb and inter–paw coordination, impaired sensitivity of the dynamic and static component of the stretch response, and a progressive degeneration of the sensory neuron and of the intrafusal fibers, which was related to increased abundance and size of lysosomes, a fragmentation of the inner and outer connective tissue capsule and a buildup of autophagic vacuoles in muscle spindles.	(45)
*GAA*–KO Mouse	Systemic.	Insertional knockout in exon 13	Satellite cells maintain regenerative capacity but fail to repair disease–associated muscle damage in mice.	(46)
*GAA*–KO mouse	Systemic.	Insertional knockout in exon 13	Lysosomal glycogen accumulation was observed in the smooth muscle of both the bronchi and the trachea in GAA–/– mice. Furthermore, GAA–/– mice had hyporesponsive airway resistance and bronchial ring contraction to the bronchoconstrictive agents methacholine and potassium chloride and to a bronchodilator. Finally, calcium signaling during bronchiolar smooth muscle contraction was impaired in Gaa–/– mice indicating impaired extracellular calcium influx.	(47)
*GAA*–KO mouse	Systemic.	Insertional knockout in exon 13	Adeno–associated virus–mediated systemic gene transfer reversed glycogen storage in all key therapeutic targets—skeletal and cardiac muscles, the diaphragm, and the central nervous system—in both young and severely affected old *GAA*–knockout mice. AAV9 vector encoding a chimeric human GAA protein was used in this study.	(48)
*GAA*–KO mouse	Systemic.	Insertional knockout in exon 13	A small molecule inhibitor of glycogen synthase 1 reduces muscle glycogen and corrects cellular pathology in *GAA*–KO Mouse.	(49)
*GAA*–KO mouse	Systemic.	Insertional knockout in exon 13	A novel Centyrin protein–short interfering ribonucleic acid conjugate targeting CD71 significantly reduced *GYS1* protein expression, glycogen synthase enzymatic activity, and glycogen levels in muscle.	(50)
*GAA*–KO mouse	Systemic.	Insertional knockout in exon 13	Miglustat increased the stability of both alglucosidase alfa and avalglucosidase alfa enzymes in fluorescent protein thermal shift assays. When incubated in neutral pH buffer over time, it reduced their enzymatic activity by −50%.	(51)
*GAA*^em1935C>A^ knock–in mouse	Systemic.	*GAA* c.1935C>A mutation	CRISPR–Cas9 system was used. Skeletal muscle weakness and hypertrophic cardiomyopathy was observed. Increased tissue glycogen storage, and concomitantly impaired autophagy was also observed.	(52)
*GAA*^c.1826dupA^ knock–in mouse	Systemic.	*GAA*^c.1826dupA^ knock–in mutation	Early onset of severe hypertrophic cardiomyopathy s and skeletal muscle weakness was observed. But these mice but did not experience early mortality.	(53)
hP545L *GAA* Tg/KO mouse	Systemic	a mutant form of human *GAA* (hP545L) on a *Gaa* KO background	The small molecule pharmacological chaperone AT2220 binds and stabilizes wild–type as well as multiple mutant forms of *GAA*, and can lead to higher cellular levels of GAA.	(54)
Mck–T–*GAA/Gaa^−/−^* mice	Cardiac and skeletal muscle	Mck–T–*GAA/Gaa^−/−^*	Levels of 20–30% of normal GAA activity were sufficient to clear glycogen in the heart of young *Gaa*^−/−^ mice, but not in older mice with a considerably higher glycogen load. Induction of *GAA* expression in skeletal muscle of young *Gaa*^−/−^ mice did not result in full phenotypic correction, and some muscle fibers showed little or no glycogen clearance.	(55)
*GAA*–KO mouse	Systemic.	Insertional knockout in exon 13	A very low number of AAV2/8vector particles was administered before initiation of ERT, to prevent the antibody response in GAA–knockout mice. And the efficacy of ERT was thereby enhanced.	(56)
*GAA*–KO mouse	Systemic.	Insertional knockout in exon 13	Adeno–associated virus vectors optimized for hepatic expression to deliver the *GAA* transgenes to *GAA* knockout mice. Therapeutic gene transfer to the liver rescued glycogen accumulation in muscle and the central nervous system, and ameliorated cardiac hypertrophy as well as muscle and respiratory dysfunction in the Gaa^−/−^ mice; mouse survival was also increased. Secretable GAA showed improved therapeutic efficacy and lower immunogenicity compared to nonengineered GAA.	(57)
*GAA*^−/−^*Cd*4^−/−^ mice	Systemic.	Insertional knockout in GAA exon 13 and insertional knockout in CD4 exon 5.	Live treated with adeno–associated virus vectors (AAV8) expressing secretable GAA with long–term ERT results in enhanced pharmacokinetics and uptake of the enzyme in peripheral tissues compared to ERT. Combination of gene transfer with pharmacological chaperones (1–Deoxynojirimycin and ambroxol) boosts GAA bioavailability, resulting in improved rescue of the PD phenotype.	(58)
*GAA*–KO/SCID mice	Systemic.	GAA and SCID double knockout.	GAA detection in the plasma was prolonged for at least 6 months secondary to the lack of anti–hGAA antibody production in all of the treated mice. GAA–KO/SCID mice treated with high doses of viral GAA vector demonstrated longer durations of glycogen correction in both skeletal and cardiac muscles, relative to mice injected with lower doses of the vector.	(59)
*GAA*–KO/SCID mice	Systemic.	GAA and SCID double knockout.	the AAV2/6 vector expressed high–level GAA and reduced the glycogen content of the injected muscle for 24 weeks. When a muscle–specific creatine kinase promoter was substituted for the CB promoter (AAV–MCKhGAApA), that AAV2/6 vector expressed high–level GAA and reduced glycogen content in immunocompetent GAA–KO mice.	(60)
*GAA*/Stbd1 double knock–out mice	systemic	GAA/Stbd1 double knock–out	In fasted double knock–out mice, glycogen accumulation in skeletal and cardiac muscles was not affected, but glycogen content in liver was reduced by nearly 73% at 3 months of age and by 60% at 13 months as compared with GAA knock–out mice, indicating that the transport of glycogen to lysosomes was suppressed in liver by the loss of Stbd1.	(61)
*GAA*–KO mouse	Systemic.	Insertional knockout in exon 13	Alglucosidase alfa with miglustat is more effective at reversing the primary abnormality—intralysosomal glycogen accumulation—in multiple muscles. This combination dramatically reduces autophagic buildup, a major secondary defect in the diseased muscles.	(62)
*GAA*–KO mouse	Systemic.	Insertional knockout in exon 13	Adeno–associated virus (AAV) vectors with a liver–muscle tandem promoter (LiMP) had a limited immune response to the *hGAA* transgene. This combination of capsid and promoter with improved muscle expression and specificity allowed for glycogen clearance in cardiac and skeletal muscles of Gaa^−/−^ adult mice. In neonate Gaa^−/−^, complete rescue of glycogen content and muscle strength was observed 6 months after AAV vector injection	(63)
*GAA*–KO mouse	Systemic.	Insertional knockout in exon 13	*GAA*–KO Mouse was treated with an adeno-associated viral (AAV) vector containing a muscle-specific promoter, AAV-MHCK7hGAApA, with β(2)-agonist clenbuterol and anti-CD4 mAb. This triple therapy increased both muscle strength and weight gain.	(64)
*GAA*-KO mouse	Systemic.	Insertional knockout in exon 13	A moss-derived nonphosphorylated rhGAA (moss-GAA), carrying a glycosylation with terminal *N*-acetylglucosamine residues reaches the target disease organs.	(65)
*GAA*–KO mouse	Systemic.	Insertional knockout in exon 13	Quadriceps glycogen content was significantly decreased by administration of the AAV vector expressing human GAA specifically in the liver with salmeterol. β2 receptor agonists enhanced the cation–independent mannose−6–phosphate receptor expression in muscle.	(66)
*GAA*–KO mouse	Systemic.	Insertional knockout in exon 13	An adeno–associated viral vector (AAV8) containing a unique liver–specific promoter (LSP) approached to GAA expression improved biochemical correction of GAA deficiency and glycogen accumulations at 18 weeks, and improved motor function testing including wire–hang and grip–strength testing.	(67)
*GAA*–KO mouse	Systemic.	Insertional knockout in exon 13	An adeno–associated virus (AAV) vector encoding human (h) GAA was pseudotyped as AAV8 (AAV2/8) and injected intravenously into immunodeficient GSD–II mice. High levels of hGAA were maintained in plasma for 24 weeks following AAV2/8 vector administration. A marked increase in vector copy number in the liver was demonstrated for the AAV2/8 vector compared to the analogous AAV2/2 vector.	(68)
*GAA*–KO mouse	Systemic.	Insertional knockout in exon 13	AAV–mediated transcription factor EB (*TFEB*) gene delivery ameliorates muscle pathology and function in the murine model of Pompe Disease.	(69)
*GAA*–KO mouse	Systemic.	Insertional knockout in exon 13	Satellite cells fail to contribute to muscle repair but are functional in Pompe disease GAA KO mice.	(70)
*GAA*–KO mouse	Systemic.	Insertional knockout in exon 13	Chronic leucine feeding restored basal and leucine–stimulated mTORC1 activation, while partially protecting Pompe mice from developing kyphosis and the decline in muscle mass. Leucine–treated Pompe mice showed increased spontaneous activity and running capacity, with reduced muscle protein breakdown and glycogen accumulation.	(71)
*GAA*–KO mouse	Systemic.	Insertional knockout in exon 13	A human codon–optimized *GAA* (*coGAA*) driven by a liver–specific promoter (LSP) using AAV9 is capable of promoting immune tolerance in a *GAA*(–/–) mouse model. Copackaging AAV9–LSP–coGAA with the tissue–restricted desmin promoter (AAV9–DES–coGAA) demonstrates the necessary cell autonomous expression in cardiac muscle, skeletal muscle, peripheral nerve, and the spinal cord. Simultaneous high–level expression in liver led to the expansion of GAA–specific regulatory T–cells (Tregs) and induction of immune tolerance.	(72)
*GAA*–KO mouse	Systemic.	Insertional knockout in exon 13	Incorporation of novel transcriptional cis–regulatory elements (CREs) and adeno–associated viral vector 9 (AAV9) gene transfer increased GAA protein level and *GAA* mRNA expression in different skeletal muscles, leading to GAA activity levels comparable with those of wild–type mice. Subsequently, this led to a significant decrease in glycogen accumulation and a restoration of centronuclear localization similar to those of wild–type levels	(73)
*GAA*–KO mouse	Systemic.	Insertional knockout in exon 13	There were significant differences in the abundance of 113 miRNA in serum exosomes from Pompe vs. healthy mice. Levels of miR−206, miR−133, miR−1a, miR−486, and other important regulators of muscle development and maintenance were altered in the Pompe samples.	(74)
*GAA*–KO mouse	Systemic.	Insertional knockout in exon 13	Electron microscopy studies at the genu of the corpus callosum revealed glycogen accumulation, an increase in nerve fiber size variation, a decrease in the g–ratio (axon diameter/total fiber diameter), and myelin sheath decompaction.	(75)
*GAA*–KO mouse	Systemic.	Insertional knockout in exon 13	GAA KO mice demonstrated significantly decreased longitudinal and transverse conductivity, increased Cole–Cole parameters.	(76)

This table provides a comprehensive overview of various genetically engineered mouse models used in Pompe disease research, detailing the targeted organs/tissues, specific genetic modifications, key pathological and therapeutic findings, and corresponding references. These models have been instrumental in elucidating disease mechanisms and testing therapeutic interventions.

**Table 4:** Extended summary of iPSC models in Pompe disease research.

**Model type**	**Target cell/Tissue**	**Mutation/Background**	**Main finding**	**Reference**
iPSC–derived neurons	Neurons	Patient–derived iPSCs (Pom–iPSCs)	Neurons recapitulate disease phenotypes. Identified ebselen, wortmannin, and PX−866 as compounds that enhance GAA activity in neurons and *in vivo*.	(77)
Isogenic murine GAA–KO cell lines	Muscle cells	Isogenic murine lines mimicking severe patient mutations	GAA–KO cells lack activity, show increased autophagy/glycogen, and downregulated CI–MPRs. The IFG chimera best restores GAA activity and normalizes p62/CI–MPRs. *In vivo*, AAV–IFG achieves cross-correction in heart.	(78)
Patient dermal fibroblasts	Fibroblasts	Fibroblasts harboring nonsense variants (c.2227C>T, c.2560C>T, c.2608C>T)	Adenine base editing achieves high deamination, restores GAA expression/activity to normal range, and reduces lysosomal burden in edited cells.	(79)
Lentiviral HSPC gene therapy model	Hematopoietic stem and progenitor cells (HSPCs)	Murine Pompe model	Lentiviral-mediated HSPC gene therapy corrects heart/muscle function and reduces glycogen accumulation after 6 months in young mice.	(80)
iPSC–derived Neural Stem Cells (NSCs)	Neural stem cells	Patient iPSC–derived NSCs	Pompe NSCs exhibit glycogen accumulation, increased lysosomal staining, and lipid buildup. rhGAA reduces lysosome size; HP–β-CD + δ-tocopherol significantly reduces phenotypes.	(81)
iPSC lines	Pluripotent stem cells	Fibroblasts from two compound heterozygous PD patients	Established iPSC lines from patients for evaluating genotype–specific therapies.	(82)
iPSC line	Pluripotent stem cells	PBMCs from an infant with compound mutations R608X E888X	Generated iPSC line expressing pluripotency markers, with trilineage potential, carrying the mutations, and a normal karyotype.	(83)
iPSC–derived Cardiomyocytes (iCMs)	Cardiomyocytes	PD patient–specific iPSC–derived iCMs	PD–iCMs show disease features: low GAA activity, glycogen accumulation, hypertrophy. Defective mitochondria (reduced number, impaired respiration, elevated ROS) underlie pathology. rhGAA improves mitochondrial function.	(84)
iPSC–derived cardiomyocytes	Cardiomyocytes	Healthy vs. inherited disease models	Proliferative responses showed genetic neutrality: comparable expansion and Ki67+/cTnT+ ratios between healthy and disease iCMs.	(85)
iPSC lines	Pluripotent stem cells	PBMCs from three GSDII patients	Patient–derived iPSC lines can be used to study pathology and evaluate therapies.	(86)
Lentiviral HSPC gene therapy model	Hematopoietic stem and progenitor cells (HSPCs)	Murine model of Pompe disease	Lentiviral vector–mediated HSPC gene therapy has been proposed as a next–generation approach for treating Pompe disease. This study demonstrates the potential of lentiviral HSPC gene therapy to reverse the pathological effects of Pompe disease in a preclinical mouse model.	(87)
iPSC lines	Pluripotent stem cells	From IOPD (homozygous c.307T>G) and LOPD (heterozygous c.−32–13T>G/c.1716C>G) patients	iPSC lines displaying pluripotent markers, normal karyotype, and trilineage differentiation capacity, representing infantile and adult–onset forms.	(88)

This table summarizes the characteristics and applications of induced pluripotent stem cell (iPSC) models derived from Pompe disease patients, including the cell types differentiated, the genetic background/mutations, main findings from studies using these models, and relevant references. These patient–specific cellular models enable the study of disease mechanisms and drug screening in a human context.

## Economic burden of Pompe disease

Pompe disease management imposes substantial economic burden, predominantly driven by ERT costs. Analysis of the French national healthcare database revealed mean annual healthcare costs of €232,117 for IOPD and €358,768 for LOPD in 2022 (24). Over 90% of LOPD costs were hospital-incurred, with ALG acquisition comprising approximately 75% of total expenses. Ventilator dependence and female sex were independent cost drivers. Patients averaged 81.9 medical/paramedical consultations and 49 hospitalization days annually (24).

Steiner et al. (25) analyzed US. data (MarketScan Databases). Among 105 ERT-treated patients, mean total all-cause costs over 2.5–2.7 years were $950,380 for IOPD and $1,857,823 for LOPD. Outpatient and ERT-related costs were primary drivers, with mean all-cause outpatient ERT costs of $308,421 (IOPD) and $778,190 (LOPD). IOPD patients had higher inpatient and supportive care utilization, while LOPD patients had higher outpatient service costs (25).

This high-cost background raises cost-effectiveness questions. A modeling study examining the UK's NICE framework highlighted a fundamental challenge: when standard care (ALG) is profoundly cost-ineffective, it distorts valuation of new technologies. A hypothetical curative gene therapy was valued at over £4 million vs. current ERT, but only £629,392 when appropriately “re-anchored” against best supportive care, indicating perceived value derives from displacing costly chronic treatment rather than intrinsic health gains (26).

## Mortality and comorbidities

A French Pompe Registry study of 60 deceased LOPD patients provided cause-of-death insights. Disease-related causes accounted for 46.7% of deaths, primarily respiratory failure (50%) and pulmonary infections (25%). Malignant neoplasms were the most common non-disease-related cause (28.3%). Patients dying from disease-related causes had significantly lower FVC (38% vs. 54.7%) and longer median delay in ERT initiation (8 vs. 1 year). The study documented high comorbidity prevalence: hypertension (42.6%), chronic constipation (24.1%), and dysphagia (22.2%) (27).

Understanding mortality patterns is crucial for improving patient management. A French Pompe Registry retrospective study analyzing 60 deceased LOPD patients provided detailed cause-of-death insights (27). Disease-related causes accounted for 46.7% of deaths, primarily respiratory failure (50% of disease-related deaths) and pulmonary infections (25%). Malignant neoplasms were the most common non-disease-related cause (28.3% of all deaths). Patients dying from disease-related causes had significantly lower FVC (38% vs. 54.7%) and longer median delay in ERT initiation after diagnosis (8 vs. 1 year) compared to those dying from unrelated causes (27). Respiratory function is thus a key prognostic factor, emphasizing early treatment importance. The study documented high comorbidity prevalence: hypertension (42.6%), chronic constipation (24.1%), and dysphagia (22.2%) (27).

## Cardiac conduction abnormalities: an emerging long-term complication

As ERT significantly extends the lifespan of IOPD patients, previously unrecognized or rare long-term complications are emerging. A critical case report by Akagi et al. (28) highlights complete atrioventricular block (cAVB) as a life-threatening arrhythmic event in a long-term survivor.

The report describes an 18-year-old male with IOPD, diagnosed at 6 months of age and successfully treated with ERT since 8 months of age, leading to remarkable improvement of his initial severe cardiomyopathy. Despite chronic ERT, he presented acutely with extreme bradycardia and cAVB, necessitating emergency temporary pacing and subsequent permanent biventricular pacemaker implantation (28). This case underscores that ERT, while life-saving for cardiomyopathy, may not fully protect against the progression of conduction system disease. This cardiac phenotype is particularly concerning given that mitochondrial structural and functional damage is increasingly recognized as a key pathogenic mechanism in PD complications.

The pathophysiology of conduction abnormalities in Pompe disease is attributed to glycogen accumulation within cardiac conduction cells, leading to cellular enlargement which can initially accelerate conduction (manifesting as a shortened PR interval, a classic ECG finding in Pompe disease) but ultimately progress to fibrosis and block (28). This aligns with previous findings in LOPD cohorts, where a small but significant proportion of patients developed high-grade AV block requiring pacemaker implantation, irrespective of ERT status (29).

This case emphasizes the critical need for lifelong, comprehensive cardiac surveillance in all Pompe disease patients, extending beyond the assessment of ventricular hypertrophy and systolic function. Regular rhythm monitoring, including Holter ECG, is essential for the early detection of progressive conduction disease, enabling timely intervention to prevent catastrophic bradyarrhythmic events (28).

## Novel biomarkers and disease pathogenesis

Conventional functional tests may be insensitive to early biological changes preceding irreversible muscle damage. Mitochondrial structural and functional impairment is increasingly recognized as an important mechanism in PD pathogenesis.

Beha et al. (30) utilized 7 Tesla ^13^C nuclear magnetic resonance (NMR) spectroscopy to non-invasively quantify muscle glycogen *in vivo*. Young, minimally affected LOPD patients had significantly elevated glycogen levels in early-degenerating muscles—lumbar paraspinals (2.2x higher) and hamstrings (1.8x higher)—while glycogen in more resilient calf muscles was normal. This suggests glycogen accumulation precedes fat replacement and clinical weakness (30). Muscle glycogen concentration thus represents a promising quantitative biomarker for assessing treatment efficacy at the molecular level, potentially enabling intervention before functional decline.

Additionally, lysosome-mediated autophagy and mitophagy dysregulation are regarded as important mechanisms in PD development, contributing to the observed increase in glial cell proportions (30). Future studies should examine relevant autophagy pathways using Western blot analysis to elucidate these mechanisms further.

## Digital health technology for sensitive motor assessment

Conventional clinical scales and timed functional tests may lack sensitivity for detecting subtle motor changes, particularly in mild or asymptomatic Pompe disease. Digital health technologies (DHTs) offer potential for detecting minute mobility alterations.

Pilotto et al. (31) conducted a case-control study evaluating DHT for detecting and quantifying mobility impairment in LOPD. Eight LOPD patients (including three young, mildly affected/asymptomatic subjects) and 52 matched controls underwent comprehensive mobility assessment using the RehaGait inertial sensor system. Compared to controls, patients showed significant alterations in walking (reduced speed, increased step time variability, shorter step length), turning (reduced angular and peak velocities), and postural transitions (prolonged standing duration, reduced maximal extension velocity) (31).

Critically, the three mildly affected/asymptomatic patients with normal clinical scale scores showed significantly increased step time variability and reduced step length compared to younger matched controls (31). This suggests digital motor metrics can detect subtle impairment before clinical manifestation (Table 5).

**Table 5:** digital health technology parameters in Pompe disease motor assessment.

**Domain**	**Specific parameters**	**Alterations in Pompe disease**	**Clinical correlation**
Gait	Step time, length, variability, asymmetry.	Increased step time variability, decreased step length.	Correlates with disease severity scales.
Turning	Duration, angular velocity, peak angular velocity.	Reduced angular velocities, prolonged duration.	Peak angular velocity correlates with clinical scores.
Postural transitions	Sit–to–stand duration, extension/flexion velocities.	Prolonged duration, reduced extension velocity.	Sit–to–stand duration correlates with clinical function.
Fatigability	Parameter changes between first/last 100 steps of 6MWT.	Minimal differences from controls.	Suggests stable deficit rather than fatigability.

This table provides a comprehensive summary of preclinical animal models used in Pompe disease research, detailing their genetic backgrounds, targeted tissues, key pathological findings (including lysosomal glycogen accumulation, autophagic buildup, mitochondrial abnormalities, and systemic dysfunction), and responses to various therapeutic interventions (including enzyme replacement therapy, gene therapy, and small molecules). Models: Various genetically engineered mouse strains (e.g., GAA-KO mice, knock–in models, tissue–specific transgenics). Outcome domains: Glycogen clearance, muscle strength and histology, cardiac function, respiratory parameters, survival, immune responses, and CNS pathology.

DHT provides richer, more objective motor function readouts than conventional assessment. In conditions like Pompe disease where early intervention modifies disease course, digital health assessment could facilitate more timely, personalized treatment decisions and enable reliable remote monitoring, potentially improving care and reducing healthcare burden (31). Longitudinal studies are needed to validate prognostic value, sensitivity to change, and role in guiding treatment decisions (Table 6).

**Table 6:** Novel therapeutic approaches in Pompe disease.

**Approach**	**Mechanism**	**Development stage**	**Key advantages**	**Limitations/Challenges**
Transferrin receptor–mediated ERT	TfR–binding fusion proteins for BBB crossing and enhanced muscle uptake.	Preclinical.	Addresses CNS manifestations; superior muscle glycogen clearance.	Immunogenicity concerns; manufacturing complexity.
Muscle–targeted AAV vectors	Engineered capsids + muscle–specific promoters for efficient gene transfer.	Preclinical.	Potential one–time treatment; reduced dose requirements.	Immune response to transgene; limited biodistribution.
Bridged bicyclic iminosugars	Enzyme stabilizers enhancing GAA stability and lysosomal delivery.	Preclinical.	Oral administration; potential adjunct to ERT.	Selectivity optimization; potential off–target effects.
Digital health technologies	Wearable sensors for continuous mobility monitoring.	Clinical validation.	Early detection; objective progression tracking.	Clinical meaningfulness thresholds; data interpretation.

This table summarizes the characteristics and applications of patient–derived induced pluripotent stem cell (iPSC) models in Pompe disease research, including the specific cell types generated (e.g., neurons, cardiomyocytes, fibroblasts), the underlying GAA mutations, main findings on disease phenotypes (e.g., glycogen accumulation, autophagy defects, mitochondrial dysfunction), and the utility of these models for evaluating genotype–specific therapies (e.g., enzyme replacement, gene editing, pharmacological chaperones). Outcome domains: GAA enzyme activity, intracellular glycogen content, cell viability and morphology, mitochondrial function, and response to therapeutic agents *in vitro*.

## Critical appraisal of evidence

A critical appraisal of the available evidence reveals several limitations and biases across key studies. For newborn screening, while umbilical cord blood shows promise, most validation studies are single-center with limited sample sizes (< 5,000), and false-positive rates due to pseudodeficiency alleles remain a challenge (10). In the ERT domain, the PROPEL trial excluded severely affected non-ambulatory patients, limiting generalizability to advanced disease stages (8, 9, 18). Variability in Hex4 reporting methods across studies complicates biomarker comparisons. Digital health studies, while innovative, often involve small sample sizes and lack longitudinal validation. Gene therapy studies remain preclinical, with immunogenicity and long-term safety yet to be established in humans. These limitations underscore the need for larger, more representative trials and standardized outcome measures.

## Limitations of current evidence and future directions

While this review highlights significant advances, several critical gaps remain. First, there is a lack of head-to-head randomized controlled trials comparing next-generation ERTs (e.g., AVA vs. Cipa+mig), forcing reliance on indirect comparisons with inherent limitations. Second, long-term data on CNS involvement in both IOPD and LOPD are inadequate, especially regarding cognitive and neurodevelopmental outcomes. Third, ethical and regulatory issues surrounding gene therapy dosing, especially in pediatric populations, require careful navigation. Fourth, barriers to implementing digital health tools—including cost, accessibility, data standardization, and clinician training—must be addressed before widespread adoption. Future research should prioritize direct comparative trials, longitudinal CNS studies, ethical frameworks for gene therapy, and pragmatic implementation studies for digital monitoring. Additionally, the consistent observation of increased glial cell proportions requires mechanistic investigation to determine whether this represents reactive gliosis, inflammatory activation, or compensatory responses to neuronal loss.

## Conclusion and future perspectives

The Pompe disease landscape is rapidly evolving, creating a more comprehensive patient journey from birth through adulthood. Umbilical cord blood validation for NBS offers a practical solution to logistical barriers, enabling earlier diagnosis, particularly of LOPD variants (10). Upon diagnosis, patients now have broader therapeutic options. Next-generation enzyme replacement therapies offer new hope, with indirect treatment comparison evidence suggesting potential AVA advantages over Cipa+mig (18), supported by COMET trial 145-week extension data confirming sustained efficacy and safety (19). Critically, a *post hoc* analysis of the COMET trial demonstrates that switching from ALG to AVA provides sustained respiratory benefit for at least 2 years, regardless of a patient's prior response to ALG, reinforcing the value of AVA in the long-term management of LOPD (21).

Concurrently, gene therapy approaches are maturing. Sellier et al. shows muscle-directed AAV vectors demonstrate efficient, low-dose treatment potential (23), with critical insights regarding anti-transgene immunogenicity management in highly specific targeting strategies.

Most notably, therapeutic frontiers are expanding to address previously untreatable CNS disease. George et al.'s (5) pioneering work on TfR-mediated ERT demonstrates, in preclinical models, effective simultaneous clearance of brain and skeletal muscle glycogen with a single biologic. This approach, coupled with novel biomarkers (e.g., CSF Hex4) and advanced imaging (Glyco-CEST/NOE MRI), opens new therapeutic possibilities.

As patient survival extends, new long-term challenges are recognized. The emergence of life-threatening cardiac conduction abnormalities, such as complete atrioventricular block in long-term IOPD survivors, underscores the necessity of lifelong, multi-system surveillance that includes arrhythmia monitoring, even in patients with stable ventricular function on ERT (28).

Amid these exciting developments, Corbett et al.'s (11) systematic review and network meta-analysis provide crucial reality checks on the current evidence base for approved ERTs. While confirming modest short-term benefits of ERTs like ALG and AVA over best supportive care in ERT-naïve patients, limited reliable evidence supports clinically meaningful outcome differences between available ERTs. The review critically highlights long-term comparative data scarcity, high bias risk in key trials, and independent research data access challenges.

Real-world evidence has clarified Pompe disease's substantial economic burden and specific mortality causes, reinforcing the critical importance of early diagnosis pathways provided by newborn screening. For disease monitoring, non-invasive biomarkers like 7T NMR spectroscopy and wearable sensor-based DHT show significant potential for early, sensitive disease activity detection at molecular and functional levels, providing earlier intervention windows (30, 31).

Looking forward, the development pipeline shows considerable promise. The prospects of curative gene therapies, highly selective oral stabilizers, advanced delivery systems (e.g., TfR-ERT), and expanded screening must be accompanied by synchronized health technology assessment and policy reform. This ensures these innovations are fairly evaluated and sustainable, equitable access models are established. The future of Pompe disease management lies in a fully integrated strategy: robust and accessible NBS, next-generation biologics and delivery systems, smart small-molecule adjuncts, advanced gene therapies with immunogenicity management, sensitive biomarkers, more robust and transparent comparative evidence bases for existing therapies, comprehensive long-term surveillance protocols for emerging complications, and supportive policy reforms—all working collectively to comprehensively improve patient outcomes from the earliest possible moment.
